# New Insights into the Interplay Between Simple Sugars and Liver Diseases

**DOI:** 10.3390/cimb47060390

**Published:** 2025-05-23

**Authors:** Simona Parisse, Erika Coltorti, Monica Mischitelli, Flaminia Ferri, Stefano Ginanni Corradini

**Affiliations:** Department of Translational and Precision Medicine, Sapienza University of Rome, Viale dell’Università 37, 00185 Rome, Italy; erika.coltorti@uniroma1.it (E.C.); monica.mischitelli@uniroma1.it (M.M.); flaminia.ferri@uniroma1.it (F.F.)

**Keywords:** fructose, sugar, liver, MASLD, diet, cirrhosis, steatosis

## Abstract

In hepatology, there is growing interest in identifying the mechanisms and risk factors underlying liver diseases with increasing incidence, with particular focus on metabolic dysfunction-associated steatotic liver disease (MASLD) and its complications. Simple sugars have been recognized as key contributors to liver injury and disease progression, not only in the context of MASLD but also beyond. As a result, numerous studies have aimed to elucidate their role in liver pathophysiology. Specifically, simple sugars have been associated with pivotal mechanisms involved in the onset of liver diseases, including inflammation, de novo lipogenesis, oxidative stress, insulin resistance, and dysbiosis with increased intestinal permeability. These mechanisms collectively contribute to a significant association between simple sugar intake and liver diseases of varying stages and severity. The scientific evidence available to date has not only clarified potential pathogenic mechanisms and clinical correlations but also led to the identification of potential therapeutic targets, encompassing both lifestyle interventions and molecular approaches. This review aims to provide a comprehensive analysis of the associations between simple sugar intake, liver injury, and liver diseases. To this end, we conducted an extensive review of the literature, selecting the most relevant and up-to-date studies on the topic.

## 1. Introduction

The significant epidemiological changes observed in the field of hepatology have prompted a discussion on the role of environmental factors in the onset and progression of chronic liver diseases. Over the past few decades, there has been a notable shift in the prevalence of liver disease etiologies. Viral etiologies have been declining, largely due to the introduction of effective antiviral therapies for HCV and widespread vaccination programs for HBV [1,2]. In contrast, metabolic etiologies have risen. Epidemiological projections indicate that, within the next 25 years, metabolic dysfunction-associated steatotic liver disease (MASLD—formerly NAFLD) will become the leading cause of liver cirrhosis and, similarly, the predominant etiology in patients with hepatocellular carcinoma (HCC) [1,3,4,5,6]. Consequently, there has been a growing need to investigate the factors driving this shift. Studies have increasingly focused on the lifestyle and dietary habits of patients, identifying simple sugars as key contributors to this emerging liver disease epidemic.

Through complex mechanisms, simple sugars can contribute both directly and indirectly to liver and systemic injury. Specifically, the cellular absorption of simple sugars leads to the formation of acetyl-CoA, which is used for ATP synthesis; when present in excess, acetyl-CoA is redirected toward de novo lipogenesis, resulting in lipid accumulation in the liver and adipose tissue. Hepatotoxic effects also arise from the generation of reactive oxygen species (ROS) and through the Maillard reaction. Moreover, it is worth highlighting that the intake of simple sugars, particularly fructose, significantly affects the composition and function of the intestinal microbiota, thereby promoting hepatic injury and fostering both hepatic and systemic inflammation. Several of these mechanisms, especially ROS generation, inflammation, and microbiota alterations, also play a key role in the development of insulin resistance, which further amplifies liver and systemic damage. This review will explore in detail these pathogenic mechanisms and their manifestation in various liver diseases [7,8,9,10,11]. The mechanisms underlying these pathological processes are intricate, involving not only the liver but also extrahepatic organs and complex systemic regulatory networks. When persistent, these alterations drive disease progression from hepatic steatosis to steatohepatitis and may ultimately lead to cirrhosis and/or the development of primary liver malignancies, particularly hepatocellular carcinoma (HCC) [1,12]. Among the interconnected pathogenetic mechanisms, the intestinal microbiota warrants special attention, not merely as a passive bystander, but as a critical player in the pathogenesis of simple sugar-induced liver damage [13]. This review aims to synthesize the most recent and relevant scientific literature, sourced from MEDLINE and PubMed, to provide an in-depth and up-to-date analysis of the role of simple sugars in liver diseases.

## 2. Definition and Classification

From a biochemical perspective, simple sugars refer to a category of molecules belonging to the carbohydrate group. Carbohydrates represent a broad biochemical class, encompassing molecules of varying complexity, all of which are fundamentally composed of carbon and water. These molecules can be simplistically represented by the biochemical formula C_n_(H_2_O)_n_ [14].

Carbohydrates are macronutrients and their primary classification is based on degree of polymerization (DP) and type of linkage (α or β), as agreed at the Food and Agriculture Organization/World Health Organization Expert Consultation in 1997. It is possible to distinguish three main groups: simple sugars (DP1–2), oligosaccharides (short-chain carbohydrates) (DP3–9) and polysaccharides (DPX10) [14].

Simple sugars comprise monosaccharides and disaccharides.

There are several definitions of monosaccharides; in general, a monosaccharide is a molecule that is not decomposable by hydrolysis and that represents a building block from which all carbohydrates are built. Furthermore, monosaccharides can be classified by the number X of carbon atoms they contain: triose [3], tetrose [4], pentose [5], hexose [6], heptose [7], and so on [14].

The most commonly considered in human health and frequently found in the diet include glucose, fructose, and galactose—they are all hexoses. Glucose is classified as an aldose due to the presence of an aldehyde group (–CHO) at the terminal position of its carbon chain, whereas fructose is a ketose, characterized by a ketone group (–CO) located within the carbon backbone. Galactose, on the other hand, is a structural epimer of glucose, differing in the configuration around a single carbon atom [15,16,17].

Disaccharides are molecules composed of two carbohydrate units linked by a glycosidic bond. The most common disaccharides include sucrose, which consists of fructose and glucose; lactose, formed by the bond between galactose and glucose; and maltose, composed of two glucose molecules [16,17]. When ingested through the diet, specific enzymes present in the intestinal lumen hydrolyze the glycosidic bond in disaccharides, converting them into monosaccharides to facilitate absorption.

Polysaccharides are long-chain molecules composed of multiple monosaccharide units linked together. They can be classified as homopolysaccharides when they are composed of the same building blocks or heteropolysaccharides when they are composed of different building blocks [16].

Among the most common polysaccharides are cellulose, which is composed of β-1,4 glycosidic bonds, and starch. Starch exists in two forms: amylose, the unbranched form consisting solely of α-1,4 glycosidic linkages, and amylopectin, the branched form that contains both α-1,4 and α-1,6 glycosidic bonds. Glycogen, the primary glucose storage polysaccharide in animals, is also composed of α-1,4 and α-1,6 glycosidic linkages. In both amylopectin and glycogen, the α-1,4 bonds form the linear segments of the molecule, while the α-1,6 bonds are responsible for the branching points in their structures [16,18].

From a general perspective, carbohydrates are essential for fundamental cellular functions and the regulation of the energetical homeostasis [9,18]. From a hepatological clinical perspective, simple sugars, as previously mentioned, have gained increasing attention due to their growing recognition as direct contributors to the development and progression of liver diseases [7,8,19].

## 3. Diet

Naturally occurring sugars are simple sugars abundant in commonly consumed foods like fruits and vegetables, and they are also used to produce natural sweeteners such as honey and table sugar. These natural sweeteners are attractive to consumers, who identify natural products as healthier options, so they are widely used in the sweetened food market to receive consumers who indicate them as healthier choices. Among natural sweeteners, fructose is the sweetest natural sugar, and it is abundant in fruit, agave nectar, honey, and some vegetables; however, the most widely used in several commercial beverages and baked products is glucose [16,20,21].

In the modern era, processed and industrially manufactured foods are being consumed more frequently, particularly in Western countries, where excessive sugar intake is increasingly recognized as a key factor in the rising prevalence of various lifestyle-related diseases [22].

Processed and industrially manufactured foods contain significantly higher concentrations of simple sugars compared to non-industrial foods [23]. Consequently, in Western societies, the primary dietary source of simple sugars now derives from industrial food products rather than natural food sources, with a consequent negative impact on health [10,23,24,25]. In industrially processed foods, the sweet component is utilized to improve the pleasantness of the flavor and might have an impact on products’ texture, color, preservation and caloric value [26].

The most commonly used sweeteners in the food industry are sucrose and high-fructose corn syrup (HFCS) [27]. HFCS represents a relatively inexpensive sugar source and is, therefore, widely utilized in the Western food industry due to its ease of production and low cost. It is industrially produced by enzymatically converting corn starch into mono- and disaccharides, including maltose, fructose, and glucose. In HFCS, fructose constitutes approximately 42–55% of the total sugar content [27,28].

HFCS is commonly found in a variety of food products, including beverages (both carbonated and other sugary drinks such as fruit juices), confectionery, jams, jellies, baked goods, and dairy products [10].

With increasing industrialization, the consumption of HFCS-containing products has steadily risen over the years. Large-scale studies conducted in the United States provide significant insights into this trend. Indeed, from the 1970s to the early 2000s, there was a continuous and significant increase in the consumption of sugary beverages and HFCS in the U.S. [29]. More specifically, between 1970 and 1997, the annual per capita consumption of HFCS increased from 64 g/day to 81 g/day [30]. While sucrose intake has declined over time, overall fructose consumption has simultaneously increased by approximately 25%. This indicates that the majority of dietary fructose is now derived from industrial sweeteners, including HFCS, rather than from natural sources [27,30,31].

The exponential increase in HFCS consumption correlates with the increased incidence of obesity and type 2 diabetes mellitus (T2DM), and steatosis and steatohepatitis, certain cancers like those of the liver or pancreas, and finally cardiovascular and kidney diseases [22].

Over the past few decades, due to increased public health awareness and policy initiatives, HFCS consumption has begun to decline, although it remains widespread in the population. In particular, per capita HFCS consumption in the United States began to decrease in the early 2000s, reaching 27 g/day in 2018 [27].

Another interesting category of sugars commonly found in the general population’s diet, originating either from natural sources or through artificial production, is represented by sugar alcohols. Sugar alcohols, or polyols, are compounds commonly used as sweeteners and bulking agents [32]. Naturally present in various plant-derived foods, they are considered sugar substitutes due to their lower caloric content compared to simple sugars and their minimal or absent requirement for insulin during metabolism. For this reason, they are particularly popular among individuals with diabetes, although they are often consumed unknowingly by the general population [33]. Among the main sugar alcohols is sorbitol, which is produced by the reduction of glucose, whereby the aldehyde group (–CHO) is converted into a primary alcohol group (CH_2_OH) [34]. Mannitol, another polyol, occurs naturally in fruits, vegetables, and seaweeds but can also be synthesized by hydrogenating mannose, similarly converting its aldehyde group into a primary alcohol. Xylitol, often referred to as “wood sugar” due to its natural occurrence in birch trees, can also be produced via biomass hydrolysis to xylose or through catalytic conversion from xylose [35]. It is widely used in sugar-free chewing gums and oral care products due to its dental health benefits [35,36]. Lastly, erythritol is a linear polyol composed of four carbon atoms, each bearing a hydroxyl group. It is virtually noncaloric (0.2 kcal/g, less than 5% of the energy value of sucrose), and it occurs naturally in fruits such as melons, grapes, and pears, as well as in fermented foods like wine and cheese. Uniquely among polyols, erythritol is not produced industrially through chemical reduction but rather via microbial fermentation. However, despite their potential benefits, emerging evidence indicates that excessive intake of foods containing sugar alcohols may exert detrimental effects. Specifically, the intracellular metabolism of polyols can activate pathways involved in cellular damage, promote de novo lipogenesis, and contribute to the formation of advanced glycation end-products (AGEs), several of which have been significantly associated with various pathological conditions and their complications, particularly in individuals with diabetes [37,38]. Moreover, recent studies have suggested that high consumption of these compounds may also be linked to poorer outcomes in patients with cirrhosis [39]. Therefore, while sugar alcohols may represent viable alternatives to traditional sweeteners, it is important to recognize the potential risks associated with their overconsumption that need to be further investigated and clarified.

Given the published data on the adverse effects of excessive sugar consumption, primarily linked to sugary beverages, including metabolic syndrome, visceral adiposity, hypertriglyceridemia, insulin resistance, and liver damage, the World Health Organization (WHO) has strongly recommended reducing sugar intake in both adults and children. Specifically, WHO guidelines advise limiting sugar intake to less than 10% of total daily energy consumption [27,29,40,41].

## 4. Metabolism

The metabolism of simple sugars varies among different types, and their distinct metabolic pathways explain their differing contributions to liver diseases. Simple sugars that reach the intestinal lumen through the diet, either in their free form or as a result of specific endoluminal enzymatic activity, are absorbed across the intestinal barrier and transported to the liver via the portal circulation [42,43].

Regarding glucose, it is essential to consider that it represents the main source of cellular energy. A significant oscillation of its concentration can be recorded between the fasting and postprandial states, ranging from 5 mmol/L to 8–10 mmol/L, respectively. An increase in blood glucose levels leads to a stimulation of insulin secretion. Insulin, in turn, facilitates the absorption of glucose in peripheral tissues, particularly in muscle and adipose tissue. In the intestinal lumen, glucose is transported mainly into cells via the membrane transporter GLUT2. In adipocytes, only a little part of the glucose taken up is stored as glycogen (<5%) of glucose taken up, since most of sugar uptake, stimulated by insulin, contributes to triacylglyceride (TG) synthesis by providing glycerol-phosphate as the backbone for free fatty acid esterification as well as through the de novo synthesis of free fatty acids. Therefore, at most, 50% of glucose is stored as TG [44].

It is estimated that only 15–25% of glucose metabolism occurs in the intestine and liver [45].

**Figure 1:**
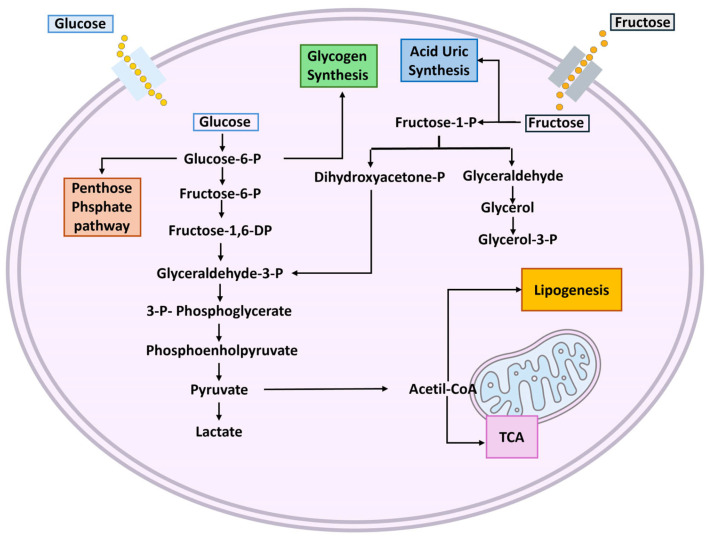
Schematic figure representing glucose end fructose intracellular metabolism. Abbreviations: TCA: tricarboxylic acids; P: phosphate; DP; diphosphate.

The intracellular metabolism of glucose begins with its phosphorylation to glucose-6-phosphate (G6P) by glucokinase. Subsequently, G6P can follow different pathways depending on cellular needs: (1) storage as glycogen for energy reserves, (2) glycolysis to produce ATP and pyruvate, or (3) the pentose phosphate pathway for nucleotide synthesis [46,47]. Glucose entering the glycolytic pathway is converted into fructose-6-phosphate and subsequently directed into glycolysis, regulated by phosphofructokinase, through its intermediate stages of fructose-1,6-bisphosphate, triose phosphate, pyruvate, and ultimately ATP + CO_2_ (Figure 1). This pathway is regulated by negative feedback, mainly controlled by high ATP and citrate concentrations [45,47]. In the liver, the primary goal of glucose metabolism is to generate glycogen reserves that can be utilized during fasting states [18,47]. When hepatic glucose levels exceed energy demands, the de novo lipogenesis pathway is activated using mainly glycolysis-derived Acetyl CoA. In this scenario, excess glucose is converted into fatty acids, which can be stored in lipid droplets within hepatocytes, incorporated into membrane structures, or assembled into very-low-density lipoproteins (VLDL) for extrahepatic transport to adipose tissue [46].

Notably, intracellular glucose may enter the polyol pathway, wherein aldose reductase catalyzes its reduction to sorbitol, which can be subsequently converted to fructose. Interestingly, this metabolic pathway, through the generation of endogenous fructose, enables the activation of multiple intracellular signaling cascades characteristic of fructose metabolism [22,37,38].

A notable distinction exists in the metabolic handling of fructose. Under basal conditions, its concentration in peripheral plasma is markedly lower than that of glucose, averaging approximately 0.04 mM. Nonetheless, following food intake, a rapid and transient elevation, up to tenfold, can be observed, with circulating fructose levels typically returning to baseline within two hours. This regulation of plasma fructose concentrations is primarily attributed to extraction process exerted by the liver, which is capable of removing up to 70% of an orally administered fructose load postprandially [48].

The main sites of fructose metabolism are the liver, intestine, and other organs and cells, such as the kidney and immune system cells [22,45,49,50].

In the intestinal lumen the sugar is transported into enterocytes via the GLUT5 membrane transporter and subsequently reaches the liver through the portal circulation [45]. A particularly interesting aspect is that, unlike glucose, fructose metabolism is unrestricted; in fact, it lacks negative feedback and is not subject to regulation by circulating hormones such as insulin [45,51].

Therefore, fructose loads can lead to large, rapid expansions in the hexose- and triose-phosphate pools, potentially providing increased substrate for all central carbon metabolic pathways, including glycolysis, glycogenesis, gluconeogenesis, lipogenesis, and oxidative phosphorylation (Figure 1) [48].

Another possible metabolic fate of fructose is its conversion into lactate, which is then released into systemic circulation and transported to other tissues [31,52].

These significant differences in the metabolism of the two primary simple sugars and the epidemiological data explain the reason why fructose is considered a key factor in the development of hepatic and cardiometabolic diseases, supporting recent dietary recommendations to limit sugar consumption published by several public health agencies, including the WHO, and the Dietary Guidelines Advisory Committee [48].

## 5. Mechanism of Liver Damage

**Figure 2:**
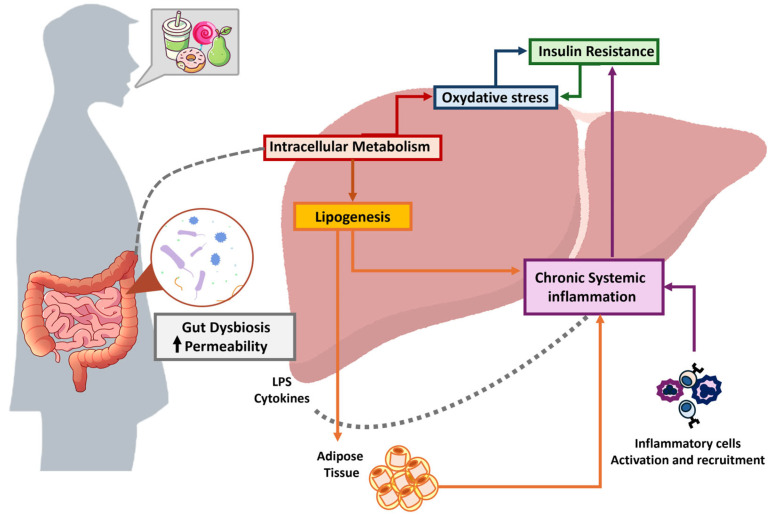
Schematic representation illustrating the interplay of damage mechanisms induced by simple sugars, with a specific focus on hepatic involvement the consumption of foods high in simple sugars can induce gut dysbiosis and increase intestinal permeability, leading to systemic inflammation and chronic hepatic inflammation. Hepatic metabolism of simple sugars may activate de novo lipogenesis, resulting in increased fatty acid synthesis and their subsequent accumulation in both hepatic and adipose tissues. This lipid overload can trigger immune activation, contributing to immune-mediated tissue injury and further promoting chronic systemic inflammation. Intrahepatic metabolism of simple sugars is also associated with the induction of oxidative stress, a key mediator of cellular damage. In this context, insulin resistance may be driven by both oxidative stress, creating a self-perpetuating cycle, and the persistent systemic inflammatory state induced by excessive simple sugar intake.

Simple sugars participate in several molecular mechanisms responsible for liver damage. Most of these mechanisms are dose-dependent and vary among individuals based on a complex interplay of genetic, epigenetic, and environmental factors. From a general perspective, sugar-mediated liver damage should be considered as a multifactorial process consisting of multiple interconnected hits, with highly complex regulatory mechanisms. In the following paragraphs, we outline some of the main mechanisms to consider when addressing liver pathologies (Figure 2).

### 5.1. Oxidative Stress

One of the key mechanisms of liver damage mediated by simple sugars is oxidative stress. A common feature among different simple sugars is that part of their metabolism is directed toward ATP production through direct or indirect activation of the glycolytic pathway. As a consequence, excessive simple sugar intake stimulates mitochondria to produce increased amounts of reactive oxygen species (ROS) [53,54].

Another mechanism that can be easily triggered by glucose or fructose overload is hyperinsulinemia and insulin resistance (see Section 5.4). When present in excess, insulin can lead to the activation of NADPH oxidase, resulting in the formation of large amounts of ROS [53,54,55].

Fructose plays an even more significant role than glucose in triggering oxidative stress mechanisms. Indeed, in addition to the aforementioned pathways, it has been described that protein fructosylation, which occurs in the presence of excess fructose and is a faster process than glucose-induced glycation, generates ROS [56]. Moreover, excessive dietary fructose intake leads to a reduction in antioxidant enzymes such as superoxide dismutase, glutathione peroxidase, and glutathione with consequent increase in ROS [57,58,59,60]. It is interesting to consider that even the consumption of HFCS has been associated with a significant increase in ROS, leading to clinical negative effects [61].

An additional interesting fructose-specific oxidative stress mechanism is linked to its metabolism via fructokinase. This enzyme is ATP-dependent and, as previously described, lacks a negative feedback regulation system. Therefore, in cases of fructose excess, the excessive ATP consumption by this enzyme leads to an increase in uric acid concentration as a byproduct of nucleotide metabolism. Interestingly, while uric acid is widely recognized as a potent extracellular antioxidant, it can exert pro-oxidant effects within cells, leading to oxidative stress. Specifically, it has been shown to activate mitochondrial NADPH oxidase, which utilizes NADPH leading to the production of ROS. In addition, uric acid can promote citrate accumulation by inhibiting aconitase, an oxidation-sensitive enzyme of the Krebs cycle, thereby enhancing de novo lipogenesis [62].

### 5.2. Inflammation

A central aspect of the mechanisms through which simple sugars exert their damaging effects at the hepatic level is inflammation. These sugars have the distinctive ability to induce a persistent systemic inflammatory state. Sugar-mediated inflammation is not limited to the liver but also involves key sites such as adipose tissue, the intestine, and, through a distinct pathway, the central nervous system [63].

This complex chronic inflammatory state involves an intricate network of cytokines, which in turn are responsible for multiple intracellular and extracellular signaling cascades. Among the main mediators of intra- and extrahepatic inflammation are C-reactive protein (CRP), peroxisome proliferator-activated receptors (PPARs), Toll-like receptor 4 (TLR-4), and most of the most common cytokines like interleukin-6 (IL-6), interleukin-4 (IL-4), and tumor necrosis factor-alpha (TNF-α) [7,63].

Studies conducted on both human subjects and animal models have reinforced existing knowledge regarding the association between simple sugar intake and elevated CRP levels [64,65,66,67,68]. Some studies indicate that, among simple sugars, fructose is linked to a notable early post-prandial increase in CRP compared to glucose [64]. However, the evidence on this topic remains debated. Notably, a compelling randomized controlled trial conducted on human found no significant differences in CRP levels after a short-term (8-day) diet high in fructose, glucose, or HFCS [65]. One possible reason for this seemingly conflicting result could be the limited duration of the high simple sugar diet. In fact, more recent studies that have analyzed patients’ habitual diets using food diaries, despite the methodological limitations, have found noteworthy associations between CRP levels and simple sugar intake [67,68].

Among simple sugars, fructose consumption has been specifically described as capable of inducing inflammation by altering intestinal permeability both through its intestinal metabolism and through specific pathways [22,45,49]. Specifically, fructose may trigger the activation of TLR4, which is naturally present in innate immune response cells and enterocytes. The activation of this receptor initiates a signaling cascade that plays a crucial role in triggering the innate immune response [69]. This process leads to the production of pro-inflammatory cytokines, including TNF-α, IL-6, and IL-1β, as well as the activation of the NLRP3 inflammasome [7,56].

Within the framework of sugar-mediated inflammation, it is essential to consider that adipose tissue, which increases due to de novo lipogenesis induced by excess simple sugars, plays a crucial role [46]. It is now well established that adipose tissue can produce and release pro-inflammatory cytokines that contribute to the systemic metabolic disorder induced by simple sugars and to liver damage [70,71].

Another noteworthy mechanism for triggering the inflammatory cascade is the direct interaction between fructose and immune cells [49]. It has been shown that a fraction of circulating fructose is taken up and directly metabolized by myeloid cells, both circulating and in bone marrow, resulting in modulation of lymphopoiesis, inflammatory cell activation, and release of pro-inflammatory cytokines [49,72,73].

A final mechanism through which simple sugars can promote chronic inflammation involves their interaction with the central nervous system. Excessive consumption of simple sugars or artificial sweeteners, particularly sucralose and fructose, has been shown to stimulate the release of corticotropin-releasing hormone (CRH) and alter the regulation of the hypothalamic–pituitary–adrenal axis [74]. CRH, in turn, stimulates the production of adrenocorticotropic hormone (ACTH), which leads to cortisol release from the adrenal glands [75]. In this way, excessive sugars establish a central modulation of stress and inflammation.

In summary, excessive simple sugar consumption unequivocally induces a systemic inflammatory state that is strongly dependent on the quantity and duration of exposure, with activation pathways that are intricate and involve multiple organs.

### 5.3. Intestinal Permeability and Gut Microbiota

As previously mentioned, another potential mechanism through which sugars can cause liver damage is the intestine.

Excessive simple sugars intake and its metabolism, which, as previously mentioned, partially occurs in enterocytes, can compromise the integrity of the intestinal barrier [22,49].

Notably, fructose appears to be the primary simple sugar responsible for these alterations, largely due to the action of fructokinase. It has been described that, in the absence of fructokinase, the alterations in intercellular junctions would not be observed [76].

These alterations are specifically characterized by the reduction in crucial enterocytes membrane proteins, including adherens junction proteins (β-catenin and E-cadherin) and tight junction proteins (claudin-1, claudin-4, ZO-1 and occludin) [58].

These changes, along with the aforementioned inflammatory mechanisms and sugar-induced shifts in gut microbiota composition, can also lead to enterocyte apoptosis [58,76].

From a general perspective, it is crucial to consider that the loss of intercellular junctions and enterocytes inevitably results in increased intestinal permeability, consequently promoting greater translocation of bacterial products into the liver through the portal circulation, thereby contributing to hepatic damage and inflammation [22,58,76,77].

Regarding changes in gut microbiota composition, although extensively studied, they are not yet fully understood. In general, the Western diet, characterized by high saturated fat and sugar intake, reduces gut microbiota diversity, promoting the growth of Firmicutes and Proteobacteria while decreasing Bacteroidetes and Fusobacteria. The reduction in Bacteroides, in particular, appears to be the alteration most strongly associated with the development of MASLD and LPS translocation [78].

In recent years, significant efforts have been directed toward understanding the relationship between gut microbiota and sugar-mediated liver damage, primarily to identify potential therapeutic targets. These may include, for example, the administration of prebiotics or probiotics, alone or in combination with omega-3 fatty acids (FA), to mitigate or prevent liver damage [79].

### 5.4. Insulin Resistance

One of the systemic mechanisms underlying metabolic syndrome and its associated liver damage is insulin resistance (IR) [80]. IR is a condition characterized by an inadequate cellular response to insulin, leading to a low sensitivity to insulin stimulation.

The damaging mechanisms induced by simple sugars, as described in previous sections, contribute to the onset of IR. In turn, IR promotes metabolic dysregulation, which exacerbates simple sugar-induced damage [53].

Specifically, oxidative stress and the subsequent production of reactive oxygen species (ROS) stimulate insulin production, and certain oxidative stress biomarkers have been associated with the presence of IR [81].

The chronic systemic inflammatory state caused by excessive simple sugar intake, characterized by elevated circulating levels of LPS, IL-1β, IL-6, and TNF-α, can inhibit insulin receptor signaling. This results in reduced insulin sensitivity in key organs such as the liver, leading to hepatic steatosis and fibrosis [53,80].

PPARα inhibition is also involved in the development of IR. This transcription factor plays a crucial role in insulin sensitivity, as demonstrated by the fact that its activation is a therapeutic target for IR treatment [82].

Endoplasmic reticulum (ER) stress, typically observed in conditions of excessive sugar intake, contributes to the IR process [80,83]. ER stress triggers the activation of JNK and NF-κB signaling pathways. Specifically, JNK induces phosphorylation of the insulin receptor substrate 1 (IRS1), negatively affecting its function [83]. Furthermore, ER stress can inhibit the Akt/PKB signaling pathway, leading to reduced insulin-stimulated glucose uptake [84].

In the setting of IR, peripheral adipose tissue plays a key role in metabolic dysregulation. IR enhances lipolytic activity, leading to elevated plasma concentrations of free fatty acids (FFAs) [85]. These excess FFAs are predominantly taken up by the liver, where they are stored as triglycerides, resulting in hepatic steatosis [86]. Once inside hepatocytes, high levels of FFAs can induce lipotoxicity by activating intracellular stress responses, including ER stress, autophagy, and the production of pro-inflammatory cytokines [87]. This lipotoxic environment, driven by sustained FFA overload, also stimulates the activation of Kupffer cells and hepatic stellate cells, fostering a pro-inflammatory and profibrotic milieu within the liver [87].

Given its high prevalence and significant impact on human health, understanding the mechanisms responsible for impaired insulin action is crucial to improving IR and elucidating the beneficial effects of insulin-sensitizing agents. Among the drugs currently widely used in this context, metformin is certainly one of the most commonly prescribed. Although its mechanism of action remains partially unclear, metformin is still considered a first-line agent for the treatment of IR. Its use enhances peripheral glucose utilization, likely through the upregulation of GLUT4 expression, and it is thought to act via both direct and indirect pathways that modulate insulin signaling [88].

## 6. Liver Disease Development and Worsening

There is increasing evidence linking excessive dietary intake of simple sugars to liver diseases. In particular, excessive consumption of simple sugars can, either alone or in combination with other stimuli, play a significant role in the onset and progression of specific liver diseases. Notably, it can lead to the accumulation of hepatic fat, resulting in MASLD. If persistent inflammatory damage occurs, it can progress to metabolic dysfunction-associated steatohepatitis (MASH). Furthermore, excessive sugar intake has also been associated with more severe clinical conditions such as cirrhosis and HCC.

### 6.1. Metabolic Dysfunction-Associated Steatotic Liver Disease and Metabolic Dysfunction-Associated Steatohepatits: Preclinical Data

The dietary consumption of simple sugars, including fructose, glucose, sucrose, and high-fructose corn syrup, has been widely associated with the development of hepatic steatosis and steatohepatitis and metabolic syndrome in experimental models [62,89,90,91,92,93].

Regarding the relationship between dietary habits and the onset of hepatic steatosis, prolonged exposure (8–24 weeks) to a high-fructose diet has been described as a factor contributing to its development [89,94].

An interesting observation to consider is that hepatic steatosis can also arise in the presence of a hypocaloric diet that is high in sugar (40%) and in the absence of weight gain [95,96].

From a qualitative perspective, comparative analyses regarding the type of sugars consumed in excess in the diet have revealed that, compared to isocaloric diets containing sucrose (glucose-fructose), a mixture of 50% free glucose and fructose is slightly more associated with fat accumulation in the liver [91].

The primary mechanism underlying fructose-induced hepatic steatosis is the stimulation of de novo lipogenesis. Key hepatic lipid metabolism enzymes affected by fructose stimulation include sterol regulatory element-binding protein 1c (SREBP-1c) and carbohydrate response element-binding protein (ChREBP). These two elements play a pivotal regulatory role in activating de novo lipogenesis and induce the expression of enzymes necessary for lipid synthesis. This metabolic regulation leads to the simultaneous activation of hepatic lipogenesis and inhibition of fatty acid β-oxidation [77,97,98,99,100].

Another mechanism through which fructose promotes lipogenesis is gene expression modulation, favoring a lipogenic profile through the negative regulation of PPARα [101,102]. Specifically, fructose enhances protein synthesis via the MTORC-RPS6KB1 pathway, promoting the accumulation of misfolded proteins and consequently inducing ER stress, which increases the expression of pro-lipogenic proteins [99]. An intriguing aspect of the effects of a fructose-enriched diet emerges from studies demonstrating that only a fructose-supplemented diet leads to a significant increase not only in the expression of enzymes involved in de novo lipogenesis metabolism but also in the upregulation of patatin-like phospholipase domain-containing protein 3 (PNPLA3) [101]. This lipase mobilizes stored triglycerides for VLDL secretion, and its polymorphism is known to be associated with lipid accumulation in hepatocytes [103].

Another critical mechanism involves systemic fatty acid transport imbalances. This metabolic shift is characterized by increased CD36 expression on hepatocyte membranes, facilitating the uptake of long-chain fatty acids from circulation, thereby enhancing hepatic lipid accumulation [77,97].

The progression of fructose-induced liver damage can lead to the transition from hepatic steatosis to steatohepatitis. This transition is mediated by oxidative stress activation and the upregulation of pro-inflammatory cytokines (see Section 5.1 and Section 5.2).

### 6.2. Metabolic Dysfunction-Associated Steatohepatitis: Data on Humans

Based on evidence obtained from animal and in vitro models, several human studies have supported the negative impact of simple sugars in the development of MASLD and metabolic syndrome.

An RCT involving four groups of 24 subjects showed that beverages containing fructose or sucrose (80 g/day for 7 weeks) increased hepatic lipogenesis compared to glucose or sugar abstention. No significant differences were observed in glycemic or insulinemic parameters, suggesting that a longer exposure time may be necessary to develop metabolic complications [104]. Similarly, another recent double-blind study reported a linear increase in hepatic steatosis, evaluated by MRI, and in insulin resistance, assessed through OGTT, in healthy individuals following dietary supplementation with HFCS beverages [105]. Mirror data were derived from the FRUITLESS study, an RCT in which the authors demonstrated that a low-fructose diet (<10 g/day for 6 weeks) significantly reduced hepatic fat content in individuals with hepatic steatosis [106]. In accordance with this finding, an RCT conducted on adolescents showed a significant reduction in hepatic fat (6.23%) and improvements in ALT and fasting insulin concentration after 8 weeks of a low-sugar diet (<3% of total daily caloric intake) [107].

An important aspect to consider is the potential relationship between the harmful effects associated with the intake of simple sugars and the dietary sources from which they are derived. Although current evidence is promising, additional research is necessary to thoroughly evaluate the impact of various food sources, further research is required to comprehensively assess the impact of different food types [108]. On this purpose, in the Maastricht Study, conducted on a population of 3981 adults (50% women), greater intrahepatic fat accumulation, evaluated via MRI, was observed when fructose was consumed in liquid form, through fruit juices and sugar-sweetened beverages, with a particularly pronounced effect in subjects with type 2 diabetes [109]. Data from a large study involving over 15,000 confirmed this association: the risk of MASLD increased by 18% for total added sugars and 20% for liquid sugars, while no significant association was found for solid sugars [110]. It has been hypothesized that fructose from whole fruits does not have the same harmful impact, thanks to the presence of fiber, antioxidants, vitamins, and flavonoids, which may counteract or modulate the steatogenic effect of fructose [111].

Lastly, Global Burden of Disease (GBD) 2017 data showed an association between the high consumption of fructose-containing sugar-sweetened beverages, (>3 g/day from carbonated beverages, sodas, energy drinks, fruit drinks) and MASLD-related mortality, further exacerbated by a diet low in Mediterranean foods (fruit, whole grains, fish, nuts) [112]. Excessive sugar consumption was particularly harmful in MASLD patients, contributing significantly to cardiovascular mortality (aHR = 2.83; 95% CI: 1.01–7.91) [113].

Overall, several human studies have demonstrated a significant association between the consumption of simple sugars and hepatic steatosis, also showing that a reduction in simple sugar intake can lead to a reversal of the condition. However, from a quantitative perspective, the studies conducted so far differ in terms of observation periods lengths, target populations, and the amounts of simple sugars investigated. As a result, it remains challenging to define the precise threshold of simple sugar intake that can be considered safe in the context of hepatology.

### 6.3. Metabolic Dysfunction and Alcohol-Related Liver Disease: Preclinical Data

Alongside the rising prevalence of MASLD, alcohol-induced liver injury is also on the rise, particularly in Western countries. Moreover, the coexistence of these two conditions is becoming increasingly frequent, to the extent that a distinct nosological entity has recently been proposed: metabolic dysfunction-associated alcohol-related liver disease (MetALD) [1,6]. MetALD is defined as a condition in which excessive alcohol intake—ranging from 140 to 350 g/week for women and 210 to 420 g/week for men—coexists with the metabolic criteria required for an MASLD diagnosis [6].

Beyond the identification of this distinct clinical entity, it has been demonstrated over time that the combined intake of alcohol and simple sugars has a synergistically deleterious effect on liver health. Supporting this concept, a preliminary in vitro study from 2012 showed that concurrent exposure of hepatocytes to glucose and alcohol exacerbates liver cell damage, mainly through oxidative stress, and significantly increases hepatocyte apoptosis [114].

Further evidence from murine models has revealed that the combination of fructose and alcohol, compared to diets rich in either alcohol or fructose alone, markedly intensifies pathogenic mechanisms. Specifically, it enhances lipogenesis, insulin resistance, inflammation, oxidative stress, and hepatic fibrosis [115,116].

An intriguing preclinical mouse model further demonstrated that short-term alcohol consumption combined with a diet high in fats and simple sugars leads to a significant increase in hepatic inflammation, characterized by the recruitment of neutrophils and macrophages, along with elevated circulating neutrophil levels. These findings suggest that the consumption of unbalanced diets rich in sugars and fats, common in Western countries, may predispose individuals to more severe liver injury when alcohol is also consumed [117].

Recently, these findings have been further substantiated using a murine model representative of MetALD. In this study, mice were subjected to a high-fat, high-sugar diet for three months, in combination with daily alcohol administration and weekly binge alcohol exposure. This regimen led to a markedly accelerated progression of liver disease. Specifically, the experimental group exhibited significantly elevated serum levels of aminotransferases and bilirubin, along with (1) enhanced cytokine secretion and increased hepatic infiltration of inflammatory cells (neutrophils and macrophages), (2) increased collagen deposition and exacerbated steatosis, and (3) impaired hepatic protein synthesis and hepatocellular dedifferentiation [118].

### 6.4. Metabolic Dysfunction and Alcohol-Related Liver Disease: Data on Humans

Current human research on the potential hepatic damage resulting from the combined intake of alcohol and simple sugars remains scarce. This is due in part to ethical limitations in experimentally replicating and analyzing the effects of these two elements when consumed together, and in part to the relatively recent recognition of the nosological category MetALD. Consequently, the existing literature predominantly focuses on the clinical outcomes of alcohol consumption in individuals with underlying metabolic risk [119,120,121,122,123].

A pivotal finding from recent large-scale cohort studies derived from the NHANES database is that individuals with MetALD exhibit a significantly higher risk of all-cause and cancer-related mortality compared to those with MASLD. These findings indicate a synergistically harmful effect of alcohol when combined with a lifestyle typical of metabolic syndrome [119,120].

An additional consideration is that alcohol consumption—especially among younger individuals—often occurs in the form of binge drinking, characterized by the rapid ingestion of large quantities of alcohol. This behavior, known to be more detrimental than chronic intake, frequently involves mixed drinks containing alcohol and sugar-sweetened beverages. Such combinations may amplify hepatotoxic effects by increasing oxidative stress, inflammation, and lipogenesis. Nonetheless, evidence exploring the interaction between binge drinking and high-sugar diets is still limited [124].

Data from Northern European population studies have shown that even low levels of alcohol intake significantly elevate the risk of liver disease and cancer in individuals with pre-existing hepatic steatosis. Moreover, binge drinking has been associated with approximately a 25% increase in the risk of developing hepatic steatosis [123].

A noteworthy study employing MRI to quantitatively assess hepatic fat content found that each additional daily unit of alcohol intake was associated with a significant increase in liver fat. Interestingly, an identical increase in hepatic fat was observed when the alcohol unit was substituted with an isocaloric sugar-sweetened beverage [122]. This supports the hypothesis that simultaneous consumption of alcohol and sugary beverages could lead to an even greater accumulation of hepatic fat.

More recently, a study assessed the risk of liver fibrosis in individuals with MASLD and low-to-moderate alcohol consumption using liver elastography. The results showed a dose-dependent increase in the prevalence of significant fibrosis with higher weekly alcohol intake [121].

In conclusion, although human data on this topic are still limited, it is reasonable to hypothesize that the combination of alcohol and a diet high in simple sugars may potentiate liver damage and have detrimental effects on overall health.

### 6.5. Cirrhosis: Preclinical Data

As previously highlighted, chronic hepatic injury mechanisms triggered by excessive simple sugar intake may ultimately culminate in liver cirrhosis. Although existing evidence on this topic remains somewhat limited and not always consistent, it is generally accepted that in the setting of chronic high sugar consumption, cirrhosis can develop through the synergistic effects of multiple hepatotoxic processes.

Among these, oxidative stress and chronic inflammation are central and mutually reinforcing contributors to liver fibrogenesis. Elevated production of ROS activates Toll-like receptors, particularly TLR4, driving Kupffer cells toward a pro-inflammatory state. This activation enhances the secretion of pro-inflammatory cytokines (TNF-α, IL-6, IL-1β), amplifying both local and systemic inflammatory responses in a self-sustaining feedback loop [125,126].

Chronic inflammation, which may also be exacerbated by increased intestinal permeability, plays a key role in hepatic stellate cell (HSC) activation. Once activated, HSCs can transdifferentiate into myofibroblasts and drive the production and deposition of type I and III collagen. The TGF-β1/SMAD3 signaling pathway is the primary driver of this process, as it (1) promotes extracellular matrix (ECM) accumulation, (2) suppresses matrix metalloproteinase activity, thereby impairing ECM degradation, and (3) enhances HSC proliferation, further perpetuating fibrosis [58,127].

Additionally, simple sugars contribute to fibrogenesis by downregulating PPAR-γ, a nuclear receptor involved in maintaining HSC quiescence, promoting fatty acid oxidation, and suppressing fibrotic signaling. Impaired PPAR-γ activity removes these regulatory constraints, thereby facilitating the progression of hepatic fibrosis [58,128].

### 6.6. Cirrhosis: Data on Humans

Current human data exploring the link between simple sugar consumption and liver cirrhosis are sparse and largely preliminary. Despite significant efforts by the scientific community to establish appropriate dietary guidelines for patients with cirrhosis, adherence to such recommendations remains low, and there is a lack of conclusive evidence regarding the role of simple sugars in the progression of the disease [129,130].

A recent observational study, which analyzed food records from patients with liver cirrhosis, highlighted a significant independent association between higher simple sugar intake and increased disease severity over time [131]. Notably, this worsening was more pronounced in individuals with elevated visceral fat, indicating that simple sugars may not only be implicated in the development of cirrhosis, as outlined previously, but may also play a role in accelerating its clinical progression, particularly in the presence of underlying metabolic dysfunction [131].

Further studies are essential to deepen our understanding of this relationship, with the aim of enhancing the evidence base and informing more effective clinical management strategies for patients with liver cirrhosis.

### 6.7. Hepatocellular Carcinoma: Preclinical Data

Due to their role in cellular metabolism, inflammation, and insulin resistance, simple sugars have frequently been investigated for their potential contribution to carcinogenesis, although results have been variable [132].

Studies specifically investigating HCC and the potential contribution of sugars to carcinogenesis remain limited and sometimes contradictory. For instance, experimental studies on HCC cell lines have shown that cancer cells may downregulate specific enzymes involved in fructose metabolism, thereby shifting their metabolic preference from fructose to glucose [133,134,135].

It is well known that cancer cells often adopt the so-called “Warburg effect”, relying predominantly on anaerobic glycolysis for ATP and lactate production even in the presence of oxygen [136].

Moreover, as described above, fructose metabolism leads to rapid ATP depletion and ROS production, which are typically associated with cellular damage and apoptosis, thereby potentially limiting tumor growth. However, under metabolic stress, tumor cells may compensate by redirecting metabolism towards the pentose phosphate pathway and mitochondrial oxidative phosphorylation. This adaptation increases antioxidant production (e.g., NADPH and glutathione) to mitigate ROS excess. This metabolic reprogramming, referred to as the “reverse Warburg effect”, may be induced by fructose [137].

Interestingly, while fructose does not seem to promote tumor growth in vitro, studies have reported that high-dose fructose administration in vivo does not inhibit tumor cell growth either [138]. This suggests that HCC cells may adapt their metabolism to utilize alternative energy sources.

In addition, tumor cells may promote their proliferation by hyperactivating the insulin receptor while simultaneously downregulating fructose-metabolizing enzymes such as aldolase B (ALDOB). Under physiological conditions, ALDOB can negatively regulate insulin signaling and de novo lipogenesis [139,140,141].

Concurrently, an upregulation of aldose reductase (AKR1B1) has been observed. When overexpressed, this enzyme diverts approximately 30% of intracellular glucose through the polyol pathway, thereby increasing endogenous fructose production [142].

This combination of events establishes a pro-tumorigenic vicious cycle characterized by enhanced glycolysis (Warburg effect), lipid accumulation, and oxidative stress.

Lastly, dietary consumption of simple sugars may indirectly contribute to hepatic carcinogenesis via intestinal sugar metabolism [143,144].

### 6.8. Hepatocellular Carcinoma: Data on Humans

With regard to the evidence linking simple sugars and HCC, it is important to first acknowledge that MASLD with or without advanced fibrosis represents a significant risk factor for this malignancy, with approximately 15% of HCC cases associated with this condition [145]. Moreover, current projections estimate that MASLD-associated HCC will become increasingly prevalent over the coming years [4]. This epidemiological trend has naturally led to increased efforts within the scientific community to identify prognostic indicators and risk factors for HCC development, particularly in the context of MASLD [12,146].

To date, there are only a limited number of human studies that have directly investigated the association between simple sugar consumption and the risk of HCC. As previously discussed, experimental studies have provided a molecular basis suggesting a potential link between simple sugars and tumorigenesis. Nevertheless, it is essential to consider that cancer development is inherently multifactorial, affected by demographics, genetic background, lifestyle, and comorbidities. Therefore, asserting a direct and unequivocal relationship between simple sugar intake and HCC onset would be overly reductive [147,148].

Some studies have reported that diets high in total sugars, glycemic load, or sugary beverage consumption are associated with an increased incidence of HCC [149,150,151].

However, a high glycemic index or load does not always correlate with a statistically significant increase in HCC risk [149,152].

Nevertheless, the findings from these various studies are undoubtedly compelling and highlight a highly relevant topic that warrants further in-depth investigation.

A recent meta-analysis aiming to synthesize the current evidence, comprising seven studies (four cohort and three case-control), found that high dietary glycemic load was associated with an increased risk of HCC among individuals with cirrhosis of viral etiology (HBV and/or HCV). In contrast, no significant association was observed in virus-negative groups [153].

In conclusion, further studies in human populations are necessary to establish whether a true causal relationship exists between simple sugar intake and HCC onset—ideally integrating clinical and experimental data within a translational research approach.

## 7. Dietary Alternatives to Simple Sugars

Sweeteners are substances of natural or artificial origin used in the food industry to replace dietary sugars, with the aim of producing low-calorie foods while maintaining a sweet and pleasant taste. Among natural sweeteners, examples include stevia, agave syrup, honey, and polyols (such as xylitol and erythritol), whereas artificial sweeteners include aspartame, sucralose, and saccharin. However, despite their reduced caloric intake, the consumption of sweeteners may negatively affect glucose and lipid metabolism, potentially leading to effects similar to those of sugars.

While studies on natural sweeteners have reported positive effects on glucose and lipid metabolism, greater concern has arisen from the use of artificial sweeteners [93].

From a general perspective, one of the primary detrimental effects of artificial sweeteners appears to be their role in inducing insulin resistance. These sweeteners can promote both de novo lipogenesis and oxidative stress, in addition to altering the gut microbiota [154]. Over time, this complex interplay of pathogenic mechanisms may lead to insulin system dysfunction, facilitating the development of insulin resistance [155].

A recent study conducted on a population of 3739 U.S. adults highlighted a significant association between the consumption of artificially sweetened beverages (ASBs) and the development of hepatic steatosis, independent of caloric intake and BMI [92]. Although the exact mechanisms underlying this association have not yet been fully elucidated, the most widely accepted hypothesis suggests that intestinal dysbiosis induced by these sweeteners acts as the primary trigger. Additionally, ASBs have been shown to play a role in reducing T-cell-mediated immune responses, which theoretically should exert a protective effect against MASLD. Given the limited number of available studies and the controversial association between artificial sweetener consumption and liver injury, further research is needed to better understand this relationship. In this regard, it is worth noting that a recent meta-analysis reported a negative association between artificial sweeteners and transaminase elevation, thereby opening the debate on the actual threshold beyond which their consumption may become harmful to humans [156].

A detailed analysis of the major artificial sweeteners has documented that sucralose, one of the most commonly used artificial sweeteners in the food industry, is poorly absorbed at the intestinal level and directly interacts with gut microbiota, promoting the proliferation of bacteria belonging to the genera Bacteroides and Clostridioides [157]. Simultaneously, this sweetener reduces bacterial metabolism of bile acids, leading to decreased activation of the farnesoid X receptor (FXR) in hepatocytes and contributing to the establishment of an inflammatory state [157].

Studies on murine models have further demonstrated that sucralose, through activation of the TIR3 receptor, induces endoplasmic reticulum stress in the liver, an increase in ROS, and upregulation of genes involved in lipogenesis (such as SREBP1, ChREBP, and others) [158].

Another well-known artificial sweetener is aspartame. The primary effects associated with this sweetener are inflammatory, including activation of the NLRP3 inflammasome, reduction in antioxidant molecule availability (such as glutathione), and increased extracellular matrix deposition in the liver with the increase in plasma ALT activity as [159,160].

It remains unclear whether long-term artificial sweetener consumption plays a role in carcinogenesis [161].

Overall, while artificial sweeteners are commonly used as an alternative to counteract the negative effects of excessive simple sugar consumption, the current literature suggests that these products may themselves act through similar pathogenic mechanisms, contributing to significant hepatic and systemic manifestations.

## 8. Conclusions

In conclusion, significant progress has been made in recent years in understanding the preclinical and clinical relationships between simple sugars and liver diseases. The available evidence supports a robust causal link between the intake of simple sugars, their metabolic pathways, and the activation of mechanisms contributing to liver injury and, consequently, to the development of chronic liver diseases of varying severity.

An important aspect to consider is that the effects of simple sugars on the liver result not only from direct mechanisms, but also from complex intracellular processes involving multiple organ systems. The impact of simple sugars should, therefore, be regarded as systemic, with a central role played by adipose tissue, the immune system, the central nervous system, the gut, and the intestinal microbiota.

With regard to liver diseases, certain associations between simple sugar intake and disease onset still require further investigation and characterization through translational studies, in order to elucidate the precise mechanisms underlying clinical observations (e.g., in the context of liver cirrhosis and HCC).

Among the objectives of future research, beyond the identification of novel molecular and microbial therapeutic targets (such as probiotics and prebiotics), it would be valuable to determine, with reasonable accuracy, the “safe” intake threshold for each type of simple sugar in relation to specific clinical conditions, in alignment with the principles of personalized and precision medicine.

## Data Availability

No new data were created or analyzed in this study.

## Abbreviations

ACTH	Adrenocorticotropic Hormone
ALDOB	Aldolase B
ASB	Artificial Sweetened Beverage
ATP	Adenosine Triphosphate
ChREBP	Carbohydrate Response Element Binding Protein
CRH	Corticotropin-Releasing Hormone
CRP	C Reactive Protein
DP	Degree of Polymerization
ECM	Extracellular Matrix
ER	Endoplasmatic Reticulum
HBV	Hepatitis B Virus
HCC	Hepatocellular Carcinoma
HCV	Hepatitis C Virus
HFCS	High Fructose Corn Syrup
IL	Interleukin
IR	Insulin Resistance
IRS1	Insulin Receptor Substrate 1
G6P	Glucose-6-Posphate
LPS	Lipopolysaccharide
MASLD	Metabolic Dysfunction-Associated Steatotic Liver Disease
MASH	Metabolic Dysfunction-Associated Steatohepatitis
METAld	Metabolic Dysfunction and Alcohol-Related Liver Disease
MRI	Magnetic Resonance Imaging
NAFLD	Nonalcoholic fatty liver disease
PNPLA3	patatin-like phospholipase domain-containing protein 3
ROS	Reactive oxygen species
SREBP-1c	Sterol regulatory element binding transcription factor 1
T2DM	Type 2 Diabetes Mellitus
WHO	World Health Organization
